# Epidaure Market–Effectiveness and transferability of a school-based intervention to improve healthy and sustainable food choices by schoolchildren: Protocol of a randomized controlled trial and qualitative study

**DOI:** 10.1371/journal.pone.0306781

**Published:** 2024-07-09

**Authors:** Aurélie Curnier, Marie Cholley-Gomez, Florian Lecêtre, Audrey Peteuil, Nicolas Meunier-Beillard, Leslie Fonquerne, Lucy Darras, Sabrina Eymard-Duvernay, Caroline Méjean, Cyrille Delpierre, Vanessa Cottet, Florence Cousson-Gélie

**Affiliations:** 1 Univ. Paul Valéry Montpellier 3, EPSYLON EA 4556, Montpellier, France; 2 Inserm UMR1231, Université de Bourgogne, CHU de Dijon, France; 3 Inserm CIC 1432, Université de Bourgogne, CHU de Dijon, France; 4 UMR1295 –Inserm, Université Paul Sabatier Toulouse III, Toulouse, France; 5 MOISA, CIRAD, CIHEAM-IAMM, INRAE, IRD, Institut Agro, Université de Montpellier, Montpellier, France; 6 Inserm UMR1231- CIC 1432, Université de Bourgogne, CHU de Dijon, France; PLoS ONE, UNITED STATES

## Abstract

**Context:**

At least 40% of cancers are linked to environmental or behavioral factors, and dietary behavior appears to be a major lever. Epidaure Market is a prevention initiative developed using a method for co-constructing health promotion initiatives and prevention programs that stratifies evidence from the scientific literature and combines it with experiential knowledge (DEVA, TPB, BCT). It promotes a sustainable diet (i.e., healthy, ecological and ethical nutrition) among 5th and 4th grade students during the crucial period of adolescence, when these behaviors are often far from the recommendations.

**Method:**

The protocol implemented was carried out in 72 middle school classes in the Montpellier and Dijon academies. The intervention included teaching sessions and a virtual supermarket game, integrated into the school curriculum and delivered by science teachers. Effectiveness is tested in a cluster randomized controlled trial with 3 evaluation times (pre- and post-intervention and 1 follow-up). The study also includes an implementation assessment, with process analysis and implementation elements, as well as a transferability assessment based on key functions (FIC model and Astaire grid).

**Expected outcomes:**

The study is still underway within the school. The primary expected outcome is a positive influence on the motives underlying food choices to move towards a sustainable diet. Secondary expectations involve changes in variables such as self-efficacy and perceived social norms, as well as an increase in knowledge about healthy eating. We also expect the qualitative approaches to provide information on the deployment process in the new territories.

**Discussion:**

The study aims not only to demonstrate the effectiveness of Epidaure Market, but also to identify the optimal conditions for its nationwide implementation in France’s middle schools. Ultimately, the initiative aims to help reduce the incidence of cancer by promoting healthier eating habits among teenagers.

## 1. Introduction

Worldwide, unhealthy eating is an evitable risk factor in the development of non-transmissible diseases such as cardiovascular disease, cancer and type 2 diabetes [1]. In France, cancer then cardiovascular disease account for the highest number of deaths [2]. In the case of cancer, the number of new cases almost doubled between 1990 and 2023 [3]. Overweight/obesity and unbalanced diet are among the main risk factors for preventable cancers, just after tobacco and alcohol [3]. In France, 19,000 new cases were attributable to overweight in 2015, and only 28% of adults ate 5 fruits and vegetables a day [3]. As a result, promoting healthy eating at all ages is a global public health priority [4] and in France, a priority area of prevention with, in particular, the ten-year cancer control strategy 2021–2030 [5] and the National Nutritrion Sante Program (PNNS 4), which aims to improve health through nutrition for the French population [6].

In addition to consequences on human health, food intake and related behaviors lie at the heart of global challenges pertaining to environmental degradation [7]. The food system, comprising the food supply chains, the food environment and consumers’ behaviors, has been estimated that just over one third of total greenhouse-gas emissions (GHGEs) stem from different procedures linked to the global food system–from production to consumption [8]. At the consumer level, the wide adoption of sustainable diets has the potential to contribute to a reduction in GHGEs and favourable health outcomes [9] Shifting towards sustainable diets requires the alignment of public health objectives, which are commonly outlined in national dietary guidelines [10, 11] with the environmental dimension of sustainability [12, 13]. The nutrition recommendations in France integrate environmental with health sustainability through specific food groups, for example limiting the intake of red and processed meats, but also highlighting aspects of production, for example increasing the consumption of foods from organic agriculture [6]. Public health strategies for children and adolescents focus on reducing the burden of non-transmissible diseases by improving diet, as eating behaviors acquired in childhood persist into adulthood [4, 14] and are more complex to change later on [15]. Some studies show that young people eat too many unhealthy foods such as fast food and high-calorie foods [16] and eat too few fruits and vegetables [17, 18]. 23% of children aged 6–17 reached the consumption benchmark of at least 5 portions of fruit and vegetables a day, and 36% of children consumed at least 125 ml of sweetened beverages (i.e. half a glass) a day [19]. Adolescents may be more sensitive than adults to peer influence in terms of risk-taking, emotionality and health behaviors [15, 20–22], but other factors may also influence their food choices. Indeed, multiple interacting factors can influence adolescents’ food choices in a complex linking individual (e.g. psychosocial, biological and lifestyle), social and environmental (e.g. family and peers), social origins and associated resources (e.g. income, time and educational level have a strong influence on eating habits), physical environment (e.g. schools, fast food outlets and other stores) and societal system (e.g. media, marketing, culture and norms) factors [23–26].

For this reason, and in order to be effective, school-based interventions seem to be the ideal place, as this is where children and adolescents spend a large part of their day and are involved in a process of learning and behavior change [27, 28]. However, they must not focus solely on a knowledge-based approach [26]. Knowledge is necessary and is one of the determinants of food choice, but it contributes relatively little to changing dietary behavior [29]. A multi-component approach, included in the school program, teacher training and parent participation seems to be more effective [27, 28, 30].

Such multi-component interventions are even more effective when they are theory-based. Indeed, implementing and evaluating theory-based interventions is recognized as an effective means of improving health-promoting behaviors [31–33], by identifying key target constructs, and selecting appropriate intervention techniques [34]. Implementing theory-based interventions is for example effective for improving physical activity among adults [35], or reducing binge-drinking among adolescents [31]. However, in this latest review and meta-analysis, Gourlan and colleagues [31] highlight that the quality of theoretical implementation was globally low for most of the studies included, and that the reciprocal link between Behavior Change Techniques [BCT, 36] and conceptual constructs remained unclear. Concerning eating behaviors among young populations, a review of the literature [30] shows that multi-strategy interventions can be effective when nutrition education program is developed with theoretical models, combined to a facilitation of the programs by school staff and teachers, parental involvement, and changes to the school food environment.

Existing interventions generally focus on obesity prevention [37, 38] physical activity [26, 39] or the food environment [40]. There is no intervention on the motives of food choice, which is realized in middle school and theoretically grounded. Previous researches about dietary behaviors have indicated that food choice motives may play a mediating role between personal norms and values and dietary behaviors [41, 42]. Indeed some types of concerns (e.g.: health, environment, etc.) may be explained by a combination of values that seems to influence dietary behaviors such as purchases and food choices and thus diet quality [41]. Several studies have showed that sustainable food motives such as such as ethics and environment, local and traditional production and health are related to healthier dietary patterns, higher consumption to fruit and vegetables and lower consumption of high-fat high sugar food, notably in young individuals [43–46]. This intervention is based on a serious game simulating a virtual supermarket (www.epidauremarket.fr).

The “Epidaure Market” intervention has been developed with a multi-disciplinary team in the Epsylon laboratory of Montpellier, using a co-construction method for health promotion actions and prevention programs that allows to stratify evidence from the scientific literature, and combine these data to experiential knowledge [DEVA method, 47]. Selected final variables integrated, among others, constructs of the theory of planned behavior [TPB, 48], one of the most widely tested models of the factors influencing health-related behaviors [49]: self-efficacity, prescriptive and descriptive social norms, attitudes, awaiting results, skills in nutrition choices, knowledge of the environmental and health impact of nutrition, and of marketing strategies. Based on an online tool [50] linking behavior change techniques and mechanisms of action, theorical constructs and relevant factors were operationalized in appropriate BCT.

Population health interventions are inherently complex. Complexity-resulting from interactions among many components parts-is a property of both the intervention and the context (or system) for which it is developed [51]. Recently, Cambon and colleagues [52, 53] have proposed the use of the notion of *interventional system* rather than intervention, in order to better take into account the interaction of contextual elements in producing the effects of an intervention. The "interventional system" is defined by the authors as: a set of interdependent human and non-human contextual agents within spatial and temporal boundaries, generating mechanistic configurations—mechanisms—that are preconditions for change in health. Process evaluations are recommended to open the "black box" of interventions in trials [54] and are considered essential for complex interventions, i.e. interventions with many potential active ingredients that are often difficult to implement [55]. As Epidaure Market is a complex intervention, a process evaluation was incorporated into the cluster randomized controlled trial.

Thus, when an intervention deployed in one context (geographical, socio-cultural, etc.) is reproduced in another context, territory, a central question concerns the conditions of its transferability. Transferability is understood as the extent to which the measured effectiveness of an applicable intervention could be achieved in another setting [56]. Transferability criteria take into account elements that belong to the intervention itself (its components and implementation conditions), but also elements from the context that can influence not only this implementation, but also the results more directly. These elements are the characteristics of the populations, the stakeholders and the context in which the intervention is implemented. Thus, the notion of transferability fully integrates that of the intervention system, in that it gives an important contribution to the effect due to the context. The main challenges of transferability studies are to increase the public health impact of an intervention evaluated as effective, by reproducing it in another context, while managing to reduce (or at least not worsen) social inequalities in health [57].

The present study describes the research protocol for a randomized controlled trial evaluating the efficacy of the intervention “Epidaure Market”, and a qualitative study evaluating its implementation and transferability. This school-based action aimed at (i) improving food choices among middle school students by acting on their motives in favor of a sustainable diet, (ii) theoretically anchored on behavior change techniques drawn from BCT, (iii) and aimed at an audience enabling the intervention to be included in the school curriculum and offered by science teachers, while involving parents with home-based work.

## 2. Method and analysis

### 2.1 Study objective and endpoints

The primary objective of the efficacy study is to assess whether the intervention influences food choice motives towards a sustainable (healthy, ecological and ethical) diet. Secondary objective will assess whether the intervention improves self-efficacy in food choices, influences social attitudes and perceptions towards food, and improves skills and knowledge in nutrition, marketing strategies and the environmental impact of food. The applicability and conditions of transferability will be assessed, in two different areas due to their disparities (Montpellier and Dijon School Academies). Hérault and Gard departments are located in the Occitanie region, a region with strong demographic growth. Whereas Nièvre, Côte d’Or and Sâone-et-Loire, Yonne departments, located in the Bourgogne-Franche-Comté region, are the 1^re^ rural region in France ahead of Bretagne with its 4 departments.

### 2.2 Study design

**Fig 1:**
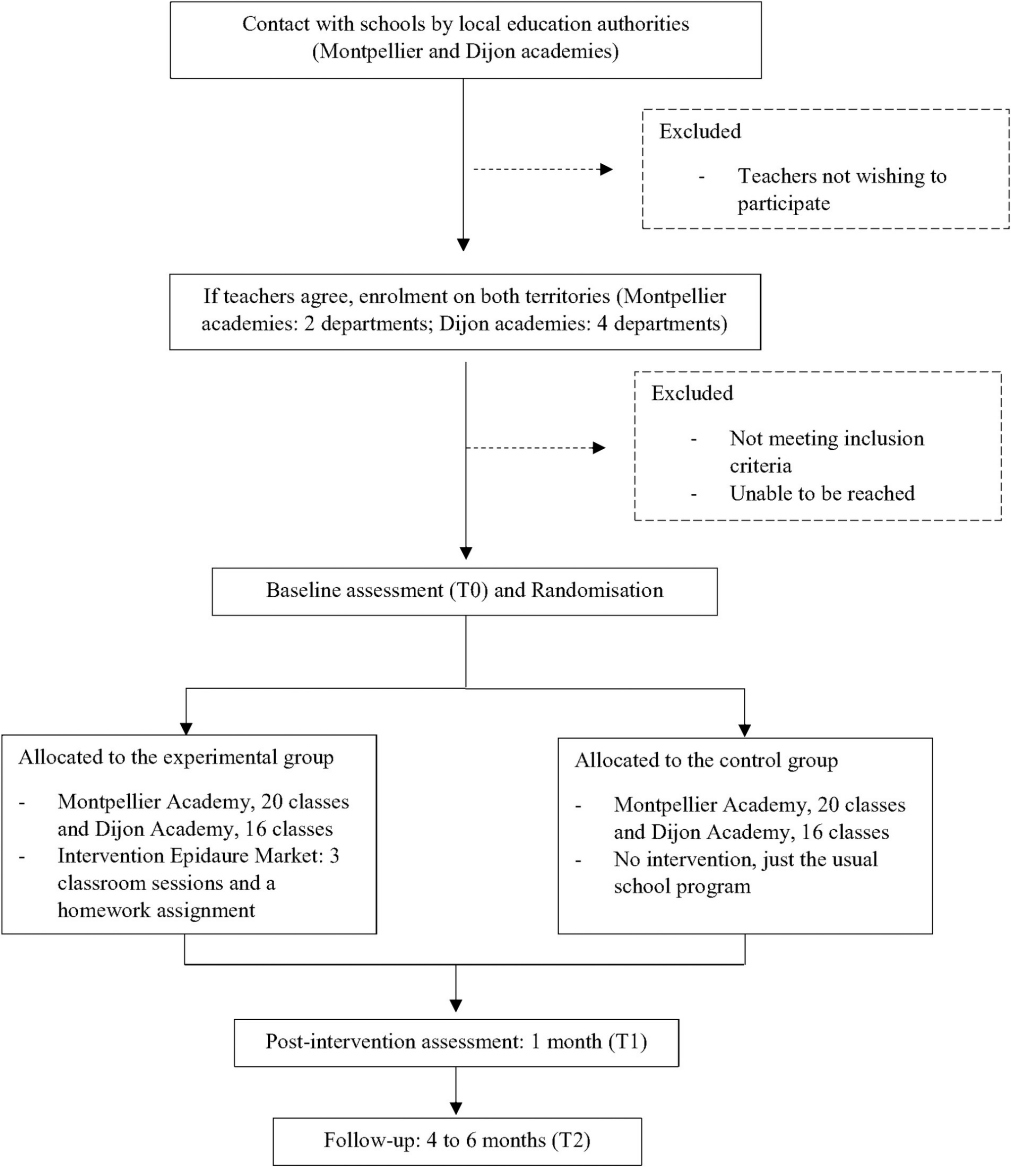
Flowchart of the Epidaure Market cluster randomized controlled trial.

Epidaure Market is a multicenter, two-arm (allocation ratio 1:1), cluster randomized controlled trial. The protocol is registered with ISRCTN registry (ISRCTN15372343). The intervention took place over three classroom sessions, organized by science teachers. Measurements are taken by the research team in both territories: before the first intervention session (T0); post-intervention (T1), one month after the end of the sessions; and a follow-up (T2), four to six months later (see Fig 1).

At the same time, a process and implementation evaluation as well as a transferability analysis are carried out. The process and implementation evaluation is based on student focus groups and semi-structured interviews with teachers, parents and school management staff and the Rectorate. Transferability is assessed using the Astaire and FIC tools. This more qualitative study was carried out after the Epidaure Market intervention.

### 2.3 Number of subjects required

The primary outcome is defined as the proportion of students who improve their motivation to healthy sustainable food choices. By assuming a proportion of 5% in the control group (due to slight variation, independent of intervention), a type I error at 5% and a variation coefficient of cluster size of 50% (because class sizes can vary between 20 and 30), 36 clusters with 25 subjects in each group (control and intervention) allow to achieve 90% power to detect a difference between 5.0% and 11.5% in the proportion of students who improve their motivation to choose good food, by varying the intra-cluster correlation between 0.02 and 0.2. A 10% attrition effect was allowed for. To compensate for this loss, more classes were recruited for each area.

### 2.4 Participants and recruitment

The design of the efficacy study is a cluster-controlled randomized trial to compare an intervention group (n = 36 classes in which the intervention is carried out) and a control group (n = 36 classes without intervention). These 72 second and third year junior secondary school classes are distributed as follows: 40 in Montpellier and 32 in Dijon. The population consists students aged between 12 and 14 years. In Occitanie, only secondary schools in the Gard and Herault departments took part in the study. Herault has 81 public secondary schools and 29 private secondary schools under contract, i.e. 110 establishments. Gard has 69 public secondary schools and 29 private secondary schools under contract, for a total of 98 establishments. At the start of the 2022 academic year, the Dijon academy (4 departments) have 158 public secondary schools and 24 private secondary schools under contract, for a total of 182 establishments. Secondary schools in the Nièvre, Côte d’Or and Soâne et Loire departments are those taking part in Epidaure Market. The last department could not be represented. To this end, all schools will be geolocated using their exact address, in order to identify the IRIS (Ilots Regroupés pour l’Information Statistique [grouped plots for statistical information]) to which they are attached. The IRIS is the basic unit for the dissemination of sub-municipal statistics, and represents a partition of the territory of these communes into "neighborhoods" with a population of around 2,000 inhabitants. The level of deprivation of the IRIS will then be assigned using the French European Deprivation Index (F-EDI) [58]. Due to school mapping, we assume that the IRIS deprivation level of the schools reflects the deprivation level of the children’s living areas, which is also assessed at the individual level. This approach has already been used in studies of the European adolescent health survey HBSC, Health Behaviour in School-Aged Children [59].

Participants for the Epidaure Market must (i) be students aged between 12 and 14 years, (ii) 5th and 4th grades, (iii) whose parents have agreed to answer the questionnaires, (iv) and have the student’s consent.

### 2.5 Randomisation

Participating classes meeting the inclusion criteria is randomized by the research teams to either the experimental group (EG) or the control group (CG). The science teacher will be the same for both experimental and control classes. It is a stratified randomization by college, with a balance between experimental and control classes. The first knowledge assessment phase (T0) will be carried out before the prevention activity. Each participant will create a participant code (based on the first three letters of their first name, followed by the first two letters of their last name, followed by the name of their middle school and class) to enable anonymous longitudinal correspondence between the 3 measurement/evaluation points.

### 2.6 Intervention

**Fig 2:**
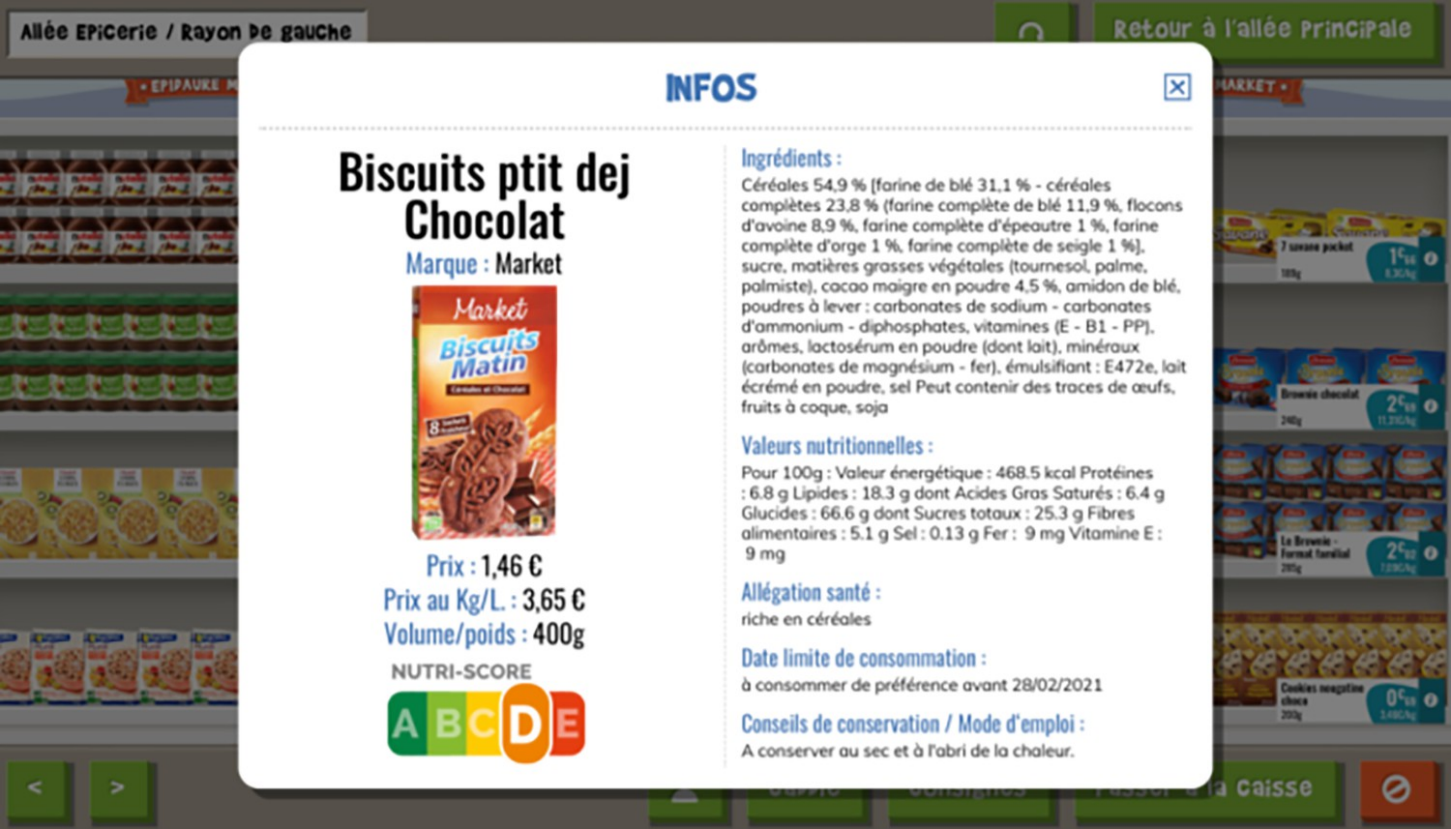
Example of the information given on food labels.

Epidaure Market is a virtual supermarket. The visual display with the different aisles, gondola heads, promotional offers, micro-announcements, etc. and the atmosphere has been designed to recall the actual experience of shopping in a supermarket and to submit the user to the current marketing methods. Users make their purchases on a screen (computer or tablet with or without touch screen) by moving around the aisles of the virtual supermarket. A cash receipt may be printed at the end of the game session to analyze the purchase choices. In addition, the receipt lists the marketing techniques used (e.g. health claims, promotions, packaging) and the price of each item, and the total cost. The first version of Epidaure Market (v1.0) was tested with 261 young people aged from 12 to 18 years. This study showed that this intervention significantly increases the knowledge of marketing techniques and promotes awareness of the influence of marketing techniques and advertising on the consumer choices, including those with potential health impacts [60]. In 2019, this tool was adapted to integrate sustainable food elements in order to increase sustainable consumption choices. Epidaure Market v2.0 is now available on tablet or computer via a site that was specifically developed for classroom use and that provides access to product labels (e.g. nutritional information, Nutri-Score, carbon footprint) (Fig 2).

**Fig 3:**
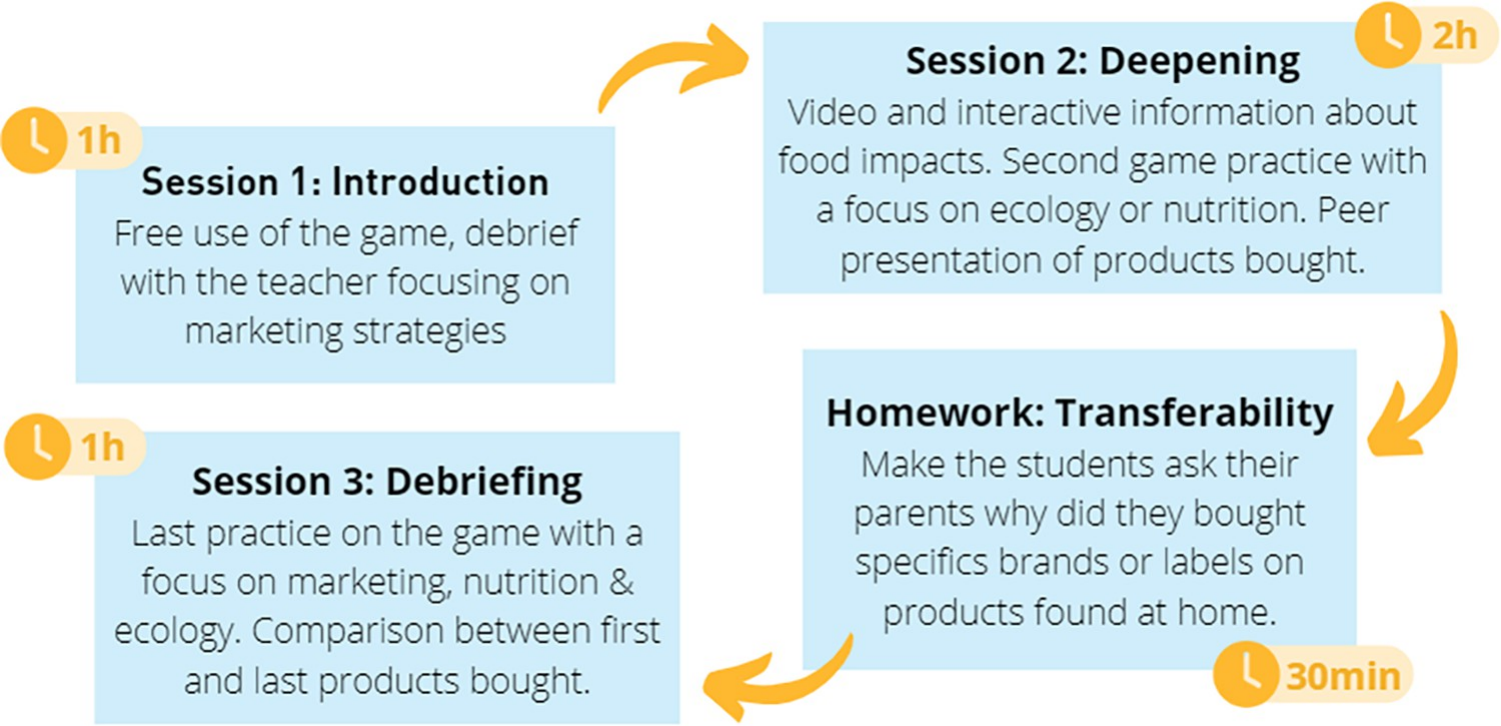
Content of the intervention.

The Epidaure Market v.2.0 intervention is a school action adapted to the science curriculum and delivered by the Life and Earth Sciences teacher for 4th and 5th graders. The intervention targets 5th and 4th graders, as food is part of the initial curriculum at this age. It is carried out over three sessions (discover, experience and become an actor) based on the serious game, with an extra-curricular session at home, that consists in an exercise involving parents to discuss familial food habits. Details of the sessions can be found in the article by Lecêtre and colleagues [61]. The content of the intervention is schematized in the Fig 3.

Each videogame session targets the students’ motives of sustainable food choices, self-efficacy, and knowledge of sustainable diet, while considering the influence of marketing and sale techniques by the food industry and supermarkets.

#### 2.6.1 Experimental group

Classes assigned to the experimental group will take part in the three Epidaure Market sessions. During these sessions, students, in pairs, shop according to a scenario predefined by the teacher (shopping for a picnic and a snack; a list of pre-tested scenarios is available on the website), with a budget of 8 euros per person. These purchases will then serve as a basis for comparison and analysis of choices during subsequent sessions. The sessions are designed around the BCTs, with the aim of influencing the motives behind teenagers’ food choices (Table 1). The full list of BCTs mobilized can be found in the development article.

**Table 1:** BCTs mobilized for each session.

	Educational Objectives	Examples of BCTs mobilized
Session 1: Introduction	1. To learn how to read price information and to learn how to compare prices; 2. To learn how to read a product label, especially in relation to the product nutritional values and sustainability; 3. To understand the Nutri-Score meaning; 4. To introduce the concepts of processed and ultra-processed food, raw and fresh products; 5. To learn how to read information about additives; 6. To raise awareness of the product origin; 7. To understand the meaning of ‘eating local’; 8. To learn how to identify the main marketing techniques.	9.1. Credibility of sources: teacher leads in a school context 4.2. Information on behavioural determinants and antecedents
Session 2: Deepening (adaptability session)	1. Learning about the different components of sustainable food 2. Marketing techniques 3. Set up 2 groups for shopping "good for health" or "good for the planet". 4. A product search task at home allows parents to be included in the intervention.	4.1. Instructions for performing the behavior 6.1. Behavior demonstration 5.1. Provide information on the health effects of the behavior 5.2. Highlighting the consequences of the behavior 8.1. Practice and repeat the behavior
Homework: Transferability	1. Knowledge transfer on everyday products 2. Introduce a discussion with parents	6.3. Information on the approval of others 8.1. Practice and repetition
Session 3: Debriefing (Knowledge Enhancement)	1. Feedback on the in-house task 2. Last buying session and comparison with session 1	6.2. Social comparison 8.1. Practice and repetition 8.7. Graduation of actions 2.2. Informing a person about their behavior

Evaluation sessions take place before the sessions (T0), one month after (T1) and 4 to 6 months after the intervention (T2). They are used to assess by questionnaires the effectiveness of the intervention, based on food choice motives, social norms, the ability to choose healthy foods and nutritional qualities or environmental impact.

Student volunteers will also take part in focus groups to discuss obstacles and levers, and assess the acceptability of the intervention. Parents will also be invited to take part in a telephone interview to answer questions on the same themes.

#### 2.6.2 Control group

Classes assigned to the control group will not have access to Epidaure Market sessions before the end of the research evaluation. They will complete all three evaluation sessions (T0, T1 and T2). Depending on their teachers, control groups could benefit of Epidaure Market intervention and use the website after the T2 evaluation session.

### 2.7 Outcomes

#### 2.7.1 Primary outcome measure

The intervention effectiveness will be assessed by comparing the proportion of students who improved their post-intervention parameters in the intervention and control groups. We chose motives for food choices because the intervention improves knowledge and motivation. Indeed, food choice motives are a key factor for changing towards sustainable behaviours [41–44, 46] and a measurement tool is available through a teen-friendly version of the questionnaire by Sautron and colleagues [62]. The questionnaire consists of 104 items divided into four categories: environmental, health and well-being, economic and miscellaneous (i.e. seasonal production, local production, etc.). The questionnaire used for this study had to be adapted to contain fewer items (28 items) and to fit in with the other questionnaires. The environmental, ethical, health and economic categories were included. All items are assessed with four-point Likert-type response scale, concerning their agreement with the stated sentence: 0-not at all agree; 1-strongly disagree; 2- quite agree; 3-strongly agree. A "don’t know" response is also possible if the student doesn’t know the word or concept evoked by the question. This questionnaire on food choice motivations is currently being validated. A factor analysis highlights a general factor: sustainable food, which will therefore be used as the primary outcome. Four secondary factors also emerge from the factor analysis and can be subdivided as follows: importance of ethics, environmental impact, locality and importance of health and absence of toxic substances. They can be used as outcomes in secondary analyses. Validation of the questionnaire will be the subject of another article.

#### 2.7.2 Secondary outcome measure

Social norms will be evaluated by self-assessment following the recommendations by Ajzen et al. [63] with six questions on perceived descriptive norms (*in general*, *my parents make healthy food purchases*) and six questions on perceived injunctive norms (*my friends think I should pay attention to the techniques that push me to buy when I shop for food*). All items are assessed with four-point Likert-type response scale, concerning their agreement with the stated sentence: 0-not at all agree; 1-strongly disagree; 2- quite agree; 3-strongly agree. A "I don’t know" response is also possible if the student doesn’t know the word or concept evoked by the question.

The Personal Efficacy Questionnaire for Eating Healthy Foods [64] will be used to assess the confidence in judgment to achieve a health outcome and confidence in the ability to choose healthy foods.

Nutrition knowledge will be assessed with a product selection task used in the ‘Opticourses’ intervention [65]. This task consists in presenting two food products that are relatively similar, but differ in some criteria, in our case nutritional qualities (Nutri-Score) or environmental impact (origin, label, season). Participants must choose which product is the best according to the available information.

#### 2.7.3 Evaluation of implementation and transferability

*Process analysis and elements of implementation*. The process analysis and conditions for implementation will be assessed using a qualitative study involving the main stakeholders (students, teachers, supervisory staff, parents). An interview guide based on the available literature has been created by the research teams to enable focus groups with pupils, semi-structured individual interviews with teachers, parents and supervisory staff (Table 2).

**Table 2:** Example of axes for process & transferability evaluation (interview guide for teacher).

Axis	Thematic inputs	Follow-up questions
***Acceptability*** *(involvement*, *relevance)*	How did the teacher find out about the project? How did he become a volunteer? What motivated him to take part in Epidaure Market? Did his motivation remain the same throughout the sessions?	What information was provided about the project and how was it transmitted? Hierarchical aspect? Had the teacher identified a need among the students? Was the project’s digital, fun aspect a lever?
***Implementation*** *(adaptation / fidelity)*	How were the sessions organised? What were the obstacles and levers, both internal and external to the intervention, during its implementation?	Adaptation/adjustment of certain sessions (form, content)? Facilitators or obstacles of a material (e.g. digital resources) or human (classroom climate) nature?

The major themes addressed will be the motivations for involvement, the facilitators and obstacles to intervention (e.g. conditions of access to supports and materials), and the conditions for implementation (acceptability, adaptation and fidelity of the intervention).

The process analysis will be carried out in the Montpellier and Dijon areas. Focus groups and semi-structured interviews will be carried out after the intervention. It is planned to carry out (i) 10 focus groups with pupils; (ii) 20 semi-structured interviews with teachers; (iii) 15 semi-structured interviews with parents of certain pupils in the experimental group, by means of a telephone interview; (iv) 10 semi-structured interviews with school management staff and the Rectorat. This forecast may be subject to change depending on access to the population in the field. Saturation, which depends on the heterogeneity and richness (quality) of the sample, as well as on the quantity of data [66], should be reached in view of the number of interviews envisaged, which is higher than recommended [67].

*Transferability analysis*. The ASTAIRE grid [68] will be used to understand the environmental factors that can influence the effects of the intervention, the methods of implementation and the means to support transfer. The ASTAIRE tool is aimed at researchers and practitioners in the field. It is made up of 2 grids: the first contains 18 criteria and the second 23, which can be classified into 4 categories: population, environment, implementation conditions, transfer support. This second tool will be used, i.e., after the implementation of the "daughter" intervention (defined as "the result of transferring an intervention that has already been tested/implemented in another context"). This grid 2 will help to evaluate a posteriori what may have generated a difference in effects between the parent intervention and the daughter intervention.

To assess the transferability and scalability of public health intervention research [52], it is necessary to identify the key elements of interventions and to take into account the context, particularly from a perspective of combating social inequalities in health. To do so, the FIC (key Functions, Implementation, Context) model [57] will be used to understand how the intervention works and to highlight the transferable theoretical elements that underline the activities implemented in a particular context, while taking into account the new context in which they can be implemented (from Montpellier to Dijon area).

The FIC model allows to structure the description of public health interventions along the lines proposed in the "Out of control" randomized trial model [69]. The model is part of the theoretical framework of population health intervention research (PHIR) [70]. The FIC model [71] has three main objectives: to improve the transferability of interventions, by distinguishing their transferable dimensions (key functions) from context-specific activities (forms); to deconstruct an intervention with the aim of describing it in detail, by identifying its key functions, which could then potentially be transferred to other contexts; to better anticipate and analyze the effects of an intervention on social inequalities in health, through this deconstruction. Key functions represent dimensions of the intervention that are potentially transferable. Forms (theoretical form as conceived by the project leaders or in case of first implementation; observed form in case of transferability which can vary under the effect of the context) refers to the practical implementation of key components through specific activities [71], which facilitate the adaptation of an intervention and enabling similar outcomes in another context. Implementation is the process from key function (recurring element) to theoretical form (adapted according to context). It is a set of activities enabling key functions to be implemented in a particular context [72]. Context influences the implementation of an intervention and affects its outcomes. It constitutes a set of interactions between actors and the environment, enabling (or not) change. It encompasses various dimensions (social, geographical, political). Thus, it contributes to structuring, modifying, facilitating and constraining the intervention, and can influence key functions and the impact of the intervention on social inequalities and to propose recommendations for taking them into account.

A qualitative multi-stage methodology is used to achieve the FIC’s objectives. This involves to participate in project meetings; reading and analyzing documents produced as part of the intervention (minutes of meetings, responses to calls for projects, documents provided to people implementing the intervention, teaching guide); carrying out bibliographical research on the intervention’s themes (sociology of childhood, socio-anthropology of food) as well as on social inequalities in health; conducting (transcribing and analyzing) focus groups or interviews with project stakeholders (members of the research team involved in the project, as well as with those implementing the intervention, mainly teachers and researchers; and, lastly, developing key functions thanks to the literature review and cross-referencing of data from all of the materials.

The FIC interviews guide is based on three main items: stage of intervention and the role of stakeholders; difficulties encountered and factors facilitating; social inequalities in health. These themes are also based on the context of expansion of intervention, the evolution of tools of intervention and their impact on inequalities, and handover between old and new research team members. Interviewees are recruited, as far as possible, taking into account the diversity of their social profile (in terms of gender, age, social class, etc.) from an intersectional perspective, and their role in the deployment of the intervention. The FIC study does not require a large number of interviews and the FIC interviews/focus groups are conducted during or after those of the implementation study.

This model will be adapted here for an analysis of the impact of interventions aiming at improving the food choices (balanced and sustainable) of middle school students, in two different French areas (Montpellier and Dijon academies), while documenting the conditions of its transferability to other regions and with other audiences (profiles of children). FIC interviews and focus groups are conducted with Montpellier research team members (N = 4) and teachers (N = 6 to 7) and with Dijon research team members (N = 3) and teachers (N = 2). A focus group could include teachers from both areas. Interview(s) with Montpellier academy member(s) (rector) could be conducted jointly by a researcher in charge of the FIC and a member of the Montpellier research team.

The ASTAIRE tool and the FIC approach are two different but complementary approaches [53, 57, 73, 74]. The FIC approach does not aim to determine the "active ingredients", i.e. the components that are essentially effective, but rather to distinguish the transferable theoretical processes which are faithful to the intervention, from the adaptable aspects of any intervention implemented. The mobilization of the ASTAIRE and FIC tools requires the collaboration of the main stakeholders involved in the design and implementation of the program; both approaches make it possible to deconstruct the intervention, to distinguish the form and key functions of it—the fixed and variable aspects- to support transferability.

### 2.8 Ethical approval and consent to participate

As part of the project entitled Epidaure Market: evaluation of the effectiveness and transferability to other schools of an intervention to improve balanced and sustainable food choices by schoolchildren, this study was approved by the research ethics committee of Université Paul Valéry—Montpellier 3 (IRB00013307- 2022–14). The electronic database used to carry out this research will comply with the recommendations of the CNIL in accordance with French law no. 78–17 of January 6 1978 on data processing, data files and individual liberties, amended by law no. 2004–801 of August 6 2004.

A letter of information, accompanied by a non-objection form to participate in the study, will be sent to the pupils’ legal representatives at least one month before the start of the intervention. This information document will explain in comprehensible terms the research objectives, design and procedures, as well as their right to refuse to participate in the study or to change their mind at any time without justification or prejudice.

Student anonymity is guaranteed by participant code. In practice, school staff will never have access to students’ answers, and the research team will never have access to students’ names.

For semi-structured interviews and focus groups, a request for consent to audio recording will be made. Anonymity will be guaranteed by assigning fictitious names, and verbatims will be anonymized if personal data is indicated during interviews. There will be no indication of the respondent’s identity.

## 3. Data analysis plan

### 3.1 Analysis of quantitative data

As the sample is made of clusters (classes) that create a correlation between the individual measures, we will consider an analysis in two stages that will use as unit of analysis the cluster (class nested in the school).

For the primary outcome measure, a cross-sectional analysis using a mixed regression model with a random constant for the class effect. More specifically, a logistic regression model will be used on the dichotomous variables. We will also measure the associated intra-class correlation coefficients. A mixed linear regression model will be used for secondary analyses, when continuous variables are involved.

Finally, additional analyses will be carried out to verify the potential effect of socio-demographic characteristics, within the same area, and between the two areas.

### 3.2 Analysis of qualitative data

#### 3.2.1 Process analysis and elements of implementation

Interview data will be analyzed by QDA Miner. A coding framework will be developed between the Dijon and Montpellier teams, and a content analysis method will be applied. This method consists of identifying content referring to themes, i.e. units of meaning that can be isolated and analyzed in a cross-cutting way, indicators allowing the inference of knowledge relating to the conditions of reception / production (inferred variables) of these statements [75]. Content analysis "breaks down, as it were, the singularity of the discourse and cuts across what, from one interview to the next, refers to the same theme"; "the identification of themes and the construction of the analysis grid are carried out on the basis of the descriptive hypotheses of the research (…). Once selected for the analysis of a corpus, the themes constitute the stable framework for the analysis of all the interviews (…)" [76, *our translation*].

The themes of interest in the interview guide, as well as new themes found in the data, will be analyzed in order to contextualize everyone’s viewpoints and experiences. When analyzing and synthesizing the interviews and focus groups, a table will be drawn up with extracts of the most important findings from the qualitative research, as well as quotes from the interviews.

#### 3.2.2 Transferability analysis

Interview data will be analyzed in two steps: by inductive content analysis method and by a coding framework based on items guide and on data from the scientific literature.

## 4. Results

Recruitment began in September 2022 and will run until June 30, 2024. The 72 classes recruited will be followed over two school years. Different classes were recruited between the 2022–2023 and 2023–2024 school years.

Focus groups and qualitative interviews began in January 2024 and will continue until the end of June 2024

## 5. Discussion

Epidaure Market is a preventive initiative for science teachers, aimed at changing the motives for food choices among teenagers in the 5th and 4th grades. The school environment is an ideal place for the implementation of health-behavior interventions. Adolescents are already in the process of learning and changing their behavior. A multi-component approach (intervention carried out in the school curriculum and by teachers) and its theoretical underpinning (DEVA method, Ajzen’s TBP, BCTs) to guide intervention design enable behavior change. Existing interventions focus on obesity prevention, physical activity or the food environment. The main objective of this study is to evaluate the effectiveness of the intervention on food choice motives.

The existing intervention designed to change eating behaviors focused on the food environment [77], in particular through the influence of product availability and accessibility [78–80]. Our intervention take account the influence of the food environment via the choice of products in the virtual supermarket.

Furthermore, little is known about the psychosocial factors which influence the behavioral change produced by these interventions [15, 26]. Thanks to the originality of its design, its theoretical foundations and the combination of objective and self-reported evaluations of food choice motivation, the Epidaure Market study is expected to add to knowledge about the effectiveness of a multi-component intervention and the psychosocial mechanisms that underlie its impact.

The school environment is the ideal place for this type of intervention [27, 28], but it has its limits. Parents’ influence on teenagers’ diet is a key factor [81, 82], but it’s difficult to reach them with this type of intervention. On the other hand, food choice motives can play a mediating role between personal norms and values and eating behaviors [41, 42]. In addition, the constraints of class time limit the content of sessions and evaluation times, which can be impacted by the conditions of the school environment and access to computer tools. At last, Epidaure Market is an action that is carried out autonomously by science teachers. Its fidelity therefore depends on the teacher’s willingness and the way in which the teaching staff is supported in setting up and deploying the intervention.

In presenting the protocol for this randomized controlled trial, we emphasize the importance of designing and implementing this trial on sound theoretical foundations related to health behavior change. Moreover, by combining the evaluation of a second territory, a transferability assessment of the intervention can be carried out with a view to future deployment at French territory level. The qualitative study will enable us to identify the levers and obstacles linked to the acceptability of the intervention, as well as to its implementation, so that we can perfect the sessions and the video game to make it accessible to as many teachers and students as possible. In a broader perspective, there is a particular interest in assessing the effectiveness and transferability of the Epidaure Market intervention, especially regarding the issues related to PHIR (Population Health Intervention Research) and the deployment of evidence-based interventions in health promotion.
